# Composite nanofiber formation using a mixture of cellulose acetate and activated carbon for oil spill treatment

**DOI:** 10.1007/s11356-022-24982-7

**Published:** 2022-12-30

**Authors:** Nehad A. Elmaghraby, Ahmed M. Omer, El-Refaie Kenawy, Mohamed Gaber, Safaa Ragab, Ahmed El Nemr

**Affiliations:** 1 Environmental Division, National Institute of Oceanography and Fisheries (NIOF), Kayet Bey, Elanfoushy, Alexandria Egypt; 2 Polymer Materials Research Department, Advanced Technology and New Materials Research Institute (ATNMRI), City of Scientific Research and Technological Applications (SRTA-City), New Borg El-Arab City, P.O. Box: 21934, Alexandria, Egypt; 3 Department of Chemistry, Faculty of Science, University of Tanta, Tanta, 31527 Egypt

**Keywords:** Cellulose acetate, Oil absorption, Machine oil, Nanofiber, Electrospinning, Activated carbon

## Abstract

Oil and organic pollutants are significant disasters affecting the aquatic ecosystem and human health. A novel nanofiber composite from cellulose acetate/activated carbon (CA/AC) was successfully fabricated by the electrospinning technique. CA/AC nanofiber composites were prepared from 10% (w/v) polymer solutions dissolving in DMA/acetone ratio 1:3 (v/v) with adding three different percentages of AC (3.7, 5.5, and 6.7%) to the total weight of CA. The prepared CA/AC nanofiber composite morphology reveals randomly oriented bead-free fibers with submicron fiber diameter. CA/AC nanofiber composites were further characterized by TGA, DSC, and surface area analysis. Water uptake was investigated for fabricated fibers at different pH. Oil adsorption was conducted in both static (oil only) and dynamic (oil/water) systems to estimate the adsorption capacity of prepared composites to treat heavy and light machine oils. The results showed increased oil adsorption capacity incorporating activated carbon into CA nanofiber mats. The maximum sorption capacity reached 8.3 and 5.5 g/g for heavy and light machine oils obtained by CA/AC5.5 (AC, 5.5%). A higher oil uptake was reported for the CA/AC composite nanofibers and showed a constant sorption capacity after the second recycles in the reusability test. Of isotherm models, the most applicable model was the Freundlich isotherm model. The result of kinetic models proved the fit of the pseudo-second-order kinetic model to the adsorption system.

## Introduction 

Petroleum hydrocarbons have drawn wide attention due to their carcinogenicity, toxicity, and mutagenicity (El Nemr 2005; Salem et al. 2014; El Nemr et al. 2016). The oil spill is considered one of the significant challenges for water pollution that can cause severe damage to both the ecosystem and human health (Khan et al. 2020; Dhaka and Chattopadhyay 2021). The most used oil/water separation methods, such as centrifugation, gravity, coalescence, and flotation methods have some limits like poor efficiency of oil separation, second pollution, high costs, and time-consuming (Wang et al. 2020). Another alternative oil/water separation technology is membrane technology with low energy consumption, hydrophobic, lipophilic, facile operation, and excellent separation efficiency (Darmanin and Guittard 2015). On the other hand, it has some drawbacks such as weak flexibility, recyclability, and durability, as well as the pores of the membrane are easily polluted or blocked by oil, and also a water membrane was formed at the surface of the membrane due to water denser than oil that hinders the contact between the oil phase and membrane (Ma et al. 2019; Satapathy et al. 2017; El Nemr and Ragab 2018). Therefore, adsorption is considered a preferable method for oil removal due to its high performance, low cost, and easy reusability (Ao et al. 2020).

A specific porous polymer-based composite nanofiber (CNFs), like low cost, high efficiency, excellent recyclability, and environmental friendliness, make them a promising adsorption material (Yue et al. 2018). Cellulose acetate nanofibers are one of the most suitable polymers for oil adsorption materials attributed to the attractive properties such as biocompatibility, small diameter of the nanofibers, non-toxicity, porosity, eco-friendly, biodegradability, and moisture retaining properties (Jatoi et al. 2019, 2020). Because of CA’s poor mechanical strength and high-temperature sensitivity, it is not favorable to use CA alone (Sabir et al. 2016, 2015; Wasim et al. 2017). These addressed issues can be overcome by addition of inorganic materials such as carbon materials, zeolite, alumina (Al_2_O_3_), silica (SiO_2_), and zirconia (ZrO_2_) which provide the possibility of additional properties that are very difficult to achieve using organic materials only (Wasim et al. 2019). Cellulose acetate nanofiber can be produced by a simple, cost-effective electrospinning technique **(**Wang and Nakane 2021). Electrospinning is an electrostatic technique that uses an applied voltage to charge the polymer particles then the nanofibers were deposited at the collector. The prepared nanofibers or sub-micron fibers are characterized by small diameter and highly porous surfacing. Because of these characteristics, electrospun fiber mats are often used in smart clothing, electrode, pharmaceutical, sensors, filtration, environmental engineering, and tissue scaffolds (Angel et al. 2020; Serag et al. 2022). Electrospinning is the method that will be used in this study to fabricate new effective eco-friendly cellulose acetate composite nanofibers with varying loads of activated carbon. These nanofibers will then be studied for their potential use in oil spill treatment. CA/C were produced using the electrospinning technology under optimal conditions into one mat. This allowed for the exponential benefits of both materials to be merged. The characterization of the generated composite nanofibers was looked into using various techniques, and the oil adsorption capacity of the nanofibers was measured using multiple adsorption settings. Additionally, the kinetics of the adsorption process of heavy machine oil (HMO) and light machine oil (LMO) as well as the reusability potential of the newly produced composite nanofibers were tested.

## Materials and methods

### Materials

ALPHA Chemie in India was the supplier of CA, which had an acetyl concentration of 29–46% and a molecular weight of 50,000. *n*-Hexane with a 95% purity level from M-TEDIA, India. Activated carbon is produced by Fisher Company. It has a microporous structure with an average pore diameter of 1.96 nm, a BET surface area of 1460 m^2^/g, and a particle size of 58.5 nm. Polyethylene glycol (PEG) is manufactured by ACROS Organic with a mass weight of 200 g/mole. Assay of 99.8% for acetone of the HPLC grade was obtained from Central Drug House, India. Merck supplies *N*,*N*-dimethylacetamide (DMAA). Ethyl alcohol is supplied by the International Company for sup and MED, Industries, located in Egypt. Exxon Mobil in Egypt (fully synthetic motor oil 5 W-50, Rally formula) supplied heavy (viscosity 32 cSt at 40 °C) and light (viscosity 5.5 cSt at 40 °C) machine oils. All of the chemicals were utilized without any further purification.

### Preparation of cellulose acetate/activated carbon solution

CA/AC composite was obtained by dissolving CA in DMA/acetone (4:1) (v/v) mixture by continuous stirring for 2 h at room temperature (24 ± 2 °C) followed by ultra-sonication overnight at room temperature to obtain a homogenous solution. The ratio of solid materials to the solvent mixture was 10% (w/v). PEG was used to facilitate the electrospinning process and overcome the solution mixture surface tension. The PEG to CA ratio was 1:1 wt/wt. Three different AC percentages (3.7, 5.5, and 6.7%) of the total cellulose acetate weight were added to CA solution (Nasir et al. 2017).

### Electrospinning of CA/AC solutions

A + 26 kV voltage was used at the injector, and –10 kV voltage was applied for the collector. A polymer solution intended for electrospinning was inserted into a syringe with a volume of 20 mL and a needle made of metal with a blunt end. At a temperature of 24 − 2 °C, the syringe pump was programmed to deliver the polymer solution at a rate of 10 mL/min with a tip-to-collector distance of 10 cm. The webs were collected on a square collector made of aluminum. After removing the nanofiber mats from the collector and washing them with ethanol and water to completely remove the PEG, the mats were dried in a vacuum oven at 50 °C for 24 h (Salihu et al. 2012; Elmaghraby et al. 2022).

### Characterization of the CA/AC nanofiber composite

The morphological characterization of the CA/AC nanofiber composites was investigated by JEOL, Model JSM 6360LA, Japan Scanning Electron Microscope (SEM) after applying a gold coating. The IMAGE-G program was used to conduct an analysis of the SEM images in order to determine the average fiber diameters. A V-100 VERTEX70 spectrophotometer was used to conduct the Fourier transform infrared spectroscopy (FTIR) examination on the samples. The thermogravimetric analyses (TGA) of the composite nanofibers were also carried out with differential scanning calorimetry (DSC) utilizing an SDT 650 simultaneous thermal analyzer from the USA. Each powdered sample was heated at a rate of 10 °C/min. In a silica crucible heated to temperatures of up to 900 °C, approximately 10 mg of each sample was paralyzed, while nitrogen gas flowed through the apparatus (Elmaghraby et al. 2022; Antunes dos Santos et al. 2021). To determine the surface area and pore size distribution of the CA/AC composite nanofibers that were formed, nitrogen adsorption–desorption isotherms were measured at 77 K using a device called BELSORP mini II. This device was developed and supplied in Japan by BEL Japan, Inc. The Brunauer–Emmett–Teller (BET) equation was used to measure the real surface area from the N_2_ adsorption isotherm, and the single point total pore volume was determined from the nitrogen sum adsorbed at 0.95 relative pressure. Both of these measurements were performed at atmospheric pressure. These two data were obtained from an isotherm depicting the adsorption of nitrogen (Elmaghraby et al. 2022; de Almeida et al. 2020).

### Water uptake capacity

The electrospun CA, CA/AC3.7, CA/AC5.5, and CA/AC6.7 composite nanofibers were further characterized by calculating their swelling and their ability to absorb water, which is essential for oil removal application. Approximately 0.1 g of CA/AC nanofiber composites was incubated at room temperature (24 ± 2 °C) for 24 h in three mediums of pH 3, 6, and 11 following the previous works of Shi et al. (2014) and Bhandari et al. (2017). The samples were softly dried using filter paper to get rid of the excess water and then weighed to obtain swelling weight (*W*_*s*_). After that, the dry weight (*W*_*d*_) was acquired by reweighing the swollen samples after drying them in an oven at 50 °C to a constant weight. This was done to get an accurate reading. Using the following Eq. (1), we could determine the product’s swelling ratio as a percentage. In this equation, *W*_*S*_ represents the weight of the swollen composite nanofibers, and *W*_*d*_ represents the weight of the dried composite nanofibers (Elmaghraby et al. 2022; Serag et al. 2018).

1 $$Selling\;ratio\;\left(\%\right)= \frac{{W}_{S}-{W}_{d}}{{W}_{d}}\times 100$$

### Oil sorption and retention capacity (static test)

The sorption test was done as 30 mL of heavy and machine oil was poured into a 250-mL beaker, then added 0.5 g of each sample of CA, CA/AC3.7, CA/AC5.5, and CA/AC6.7 composites for 1 h in room temperature (24 ± 2 °C). The composite samples were lifted, placed on a wire mesh, drained for 5 min to eliminate any loosely attached oil, and weighed. Then, the samples were elevated with free oil dripping out for 24 h to determine the oil retention capacity. All investigations were repeated 3 times, and only the mean value was reported as the final results. The oil sorption capacity (*Q*_*osc*_) (g/g) and retention capacity (*Q*_*orc*_) (g/g) were calculated according to Eqs. (2) and (3) (Elmaghraby et al. 2022; Dong et al. 2015).

2 $$Oil\;sorption\;capacity\;\left({Q}_{osc}\right)=\frac{{W}_{5}-{W}_{i} }{{W}_{i}}$$

3 $$Oil\;retention\;capacity\;({Q}_{orc})=\frac{{W}_{24}-{W}_{i}}{{W}_{5}- {W}_{i}}$$

where *W*_*i*_, *W*_5_, and *W*_24_ (g) are the initial mass of the sample before sorption, the mass of oil wetted sample at 5 min and 24 h dripping, respectively.

### Batch sorption experiments (oil water system)

An experiment series was carried out to study the sorption removal performance of the CA/AC nanofiber composites for absorbing light and heavy machine oils from the water surface. Different sorption parameters have been investigated as sorbent dosage, sorption time, and oil thickness. CA/AC nanofiber composites were placed into a 250-mL beaker with a certain amount of machine oil and 50 mL of seawater, and then they were vibrated at 200 rpm, for various interval times of 5, 10, 15, 30, and 60 min. Then the samples were removed into a wire mesh, lifted to free oil draining for 5 min and weighted. Subsequently, the composite samples were squeezed five times to eliminate the residual oil, and a small amount of hexane was added to help extract oil from the fibers. The residual liquid was centrifuged for water content determination by using a graduated centrifuge tube. The capacity of oil sorption was measured following Eq. (4), where *W*_*i*_, *W*_*w*_, and *W*_*s*_ are the initial dry weight of sorbent, the weight of adsorbed water, and the weight of saturated sorbent (water + oil + sorbent), respectively (Elmaghraby et al. 2022; Alaa El-Din et al 2017; Martins et al. 2021).

4 $${Q}_{osc}=\frac{{W}_{S}-{W}_{w}-{W}_{i}}{{W}_{i}}$$

### Reusability test

The reusability of the fabricated CA/AC composite nanofibers was investigated for static and dynamic tests. The oil-observed fibers were placed in a centrifuge to separate the absorbed oil and then washed with 3 mL *n*-hexane and centrifuge again. After washing, the fibers were dried for 24 h at 60 °C in an oven and then the regenerated composite nanofibers were utilized again in the next oil-sorption process. The static test was done as 0.5 g of regenerated fibers inserted into 30 mL of oil for 1 h at RT (24 ± 2 °C) for both LMO and HMO. Where the oil/water test was performed as 0.4 g of regenerated fibers with seawater (50 mL) and 5 mm oil thickness layer of HMO and 7-mm-oil thickness of LMO for 15 min and 1-h contact times, respectively. The recycling steps were repeated for 4 cycles, and the oil sorption capacity of the fibers after each cycle was calculated (Elmaghraby et al. 2022; Long et al. 2021).

### Adsorption isotherm studies

The absorption isotherm of the experimental data was investigated using Freundlich, Langmuir, and Tempkin isotherm models (IM) (Elmaghraby et al. 2022; El Nemr et al. 2010). The theory of Langmuir suggests that the adsorption takes place on a certain homogenous site of the adsorbent. The maximum sorption capacity was calculated according to the saturation of the monolayer on the surface of the sorbent. The linear Langmuir model form is expressed in Eq. (5) (Langmuir 1916; Longhinotti et al. 1998).

5 $$\frac{{C}_{e}}{{q}_{e}}=\frac{1}{{K}_{a}{Q}_{m}}+\frac{1}{{Q}_{m}}\times {C}_{e}$$

where *C*_*e*_ (mg/L) is the equilibrium concentration; *q*_*e*_ (mg/g) denotes the amount of oil sorbed; *Q*_*m*_ (mg/g) represents the maximum sorption capacity; *K*_*a*_ (L/mg) is the sorption equilibrium constant.

The key assumption of a Freundlich equation is that the surface has a heterogeneous composition and a non-uniform distribution of heat due to adsorption processes taking place on the surface. The Freundlich exponential equation, which postulated that the adsorbate concentration would increase as the oil concentration on the adsorbent surface increased, assumed this would be the case. From a value of 1/*n* from Freundlich equation it was clearly found that a normal Langmuir isotherm occurred at a value for 1/*n* below one, while cooperative adsorption occurred at a value for 1/*n* above one. Equation (6) expressed the linear form of the Freundlich isotherm (Freundlich 1906):

6 $$\log\;q_e=\log\;K_f+\frac1n\log\;C_e$$

where *K*_*f*_ and *n* showed the adsorption capacity of the Freundlich constant and the adsorption rate constant, respectively (Nasseh et al. 2019).

The Tempkin isotherm model supposed that the adsorption heat decreases linearly with coverage due to the interaction between adsorbent and adsorbate (Tempkin and Pyzhev 1940). The linear Tempkin model is expressed in the simplified Eq. (7) (Aharoni and Ungarish 1977; Aharoni and Sparks 1991; Wang and Qin 2005):

7 $${q}_{e}=\beta lnA+\beta ln{C}_{e}$$

where *β* = (*RT*)/*b*, *R* (8.314 J/mol K) is the universal gas constant; *T* (Kelvin) is the absolute temperature; and *b* is a constant related to the heat of adsorption (Pearce et al. 2003; Akkaya and Ozer 2005).

### Best-fit IM

To estimate the most suited IM for experimental data, the error functions were investigated for the studied IM, where the symbol *N* and *P* refer to the number of experimental data points and the number of IM parameters, respectively. The average percentage errors (APE) showed the fit between the predicted and experimental adsorption capacity data, respectively, and can be determined by the Eq. (8) (Ng et al. 2002).

8 $$APE\left(\%\right)=\frac{100}N\sum\nolimits_{i=1}^N\left|\frac{qe,\;isotherm-qe,\;calc}{qe,\;isothem}\right|\mathrm i$$

The hybrid fractional error (Hybrid) function is the most dependable error function because it accounts for low concentrations by balancing absolute deviation against fractional error and being represented as the Eq. (9) (Porter et al. 1999; Allen et al. 2003).

9 $$Hybrid=\frac{100}{N-P}\sum\nolimits_{i=1}^N\left|\frac{qe,\;isotherm-qe,\;calc}{qe,\;isothem}\right|\mathrm i$$

The chi-square error, *X*^2^ is given as the equation (El Nemr et al. 2010).

10 $$X^2=\sum\nolimits_{i=1}^N\frac{\left(qe,\;isotherm-qe,\;calc\right)^2}{qe,\;isothem}$$

The sum of the squares of the errors (*ERRSQ*) is given by the following Eq. (11) (Ng et al. 2002).

11 $$ERRSQ=\sum\nolimits_{i=1}^N{(qe,\;calc-qe,\;isotherm)}^2\mathrm i$$

The Marquart’s percentage standard deviation (*MPSD*) is given by the following Eq. (12) (Ng et al. 2002).

12 $$MPSD={\sqrt{\frac1{N-P}\sum_{i=1}^N(\frac{qe,\;calc-qe,\;isotherm}{qe,\;isotherm}})}^2\mathrm i$$

The sum of the absolute errors (*EABS*) is given by the following Eq. (13) (Ng et al. 2002).

13 $$EABS={\sum }_{i=1}^{N}\left|q e, calc- q e, isotherm\right|\mathrm{i}$$

The root mean square errors (RMS) are given by Eq. (14) (Ng et al. 2002).

14 $$RMS=100\times{\sqrt{\frac1N\sum \nolimits_{i=1}^N(1-\frac{qe,\;calc}{qe,\;isotherm}})}^2$$

### Kinetic studies

The sorption kinetics investigates the rate of oil sorption, and it is essential to find the best conditions for the batch experiments. The most common kinetics models for studying the equilibrium data are pseudo-first-order (PFO) (Lagergren 1898) and pseudo-second-order models (PSO) (Ho et al. 2005). The correlation coefficient (*R*^2^, with values close to or equal to 1) was deemed to be a measure of concordance between experimental data and the model-predicted values; the model with the relatively higher value is the one that is more appropriate to the kinetics of oil sorption. The PFO model was used to characterize the kinetic data. This model characterizes the rate of oil sorption in accordance with the number of vacant sites. The ratio of occupied adsorption sites to the total number of adsorption sites is directly related to the occupation rate (Choi et al. 2020). The PFO equation is given by Eq. (15):

15 $$\log\;\left(q_e-q_t\right)=\log\;q_e-\frac{K_1}{2.303}t$$

where, *q*_*t*_ (mg/g) and *q*_*e*_ (mg/g) represent the amount of oil on the composite at time *t* (min) and at equilibrium time, respectively, and *K*_1_ (L/min) is the adsorption rate constant of PFO.

The PSO kinetic model supposed that the adsorption takes place in the square-shaped sites, where at equilibrium, there is a relationship between the number of available adsorption sites on the absorbent and that of the occupied sites. There is a correlation between the square of the product of the number of vacant sites and the number of occupied sites and the adsorption rate (Choi et al. 2020). The PSO can be represented by Eq. (16):

16 $$\frac{t}{{q}_{t}}=\frac{1}{{K}_{2}{q}_{e}^{2}}+\frac{1}{{q}_{e}}t$$

where, *K*_2_ represented the PSO rate constant of sorption (g/mg min). Using the following Eq. (17), the PSO rate constants were utilized to obtain the initial sorption rate.

17 $$h={K}_{2}{q}_{e}^{2}$$

## Results and discussion

### *A*nalyses of CA/AC nanofiber composites

**Fig. 1:**
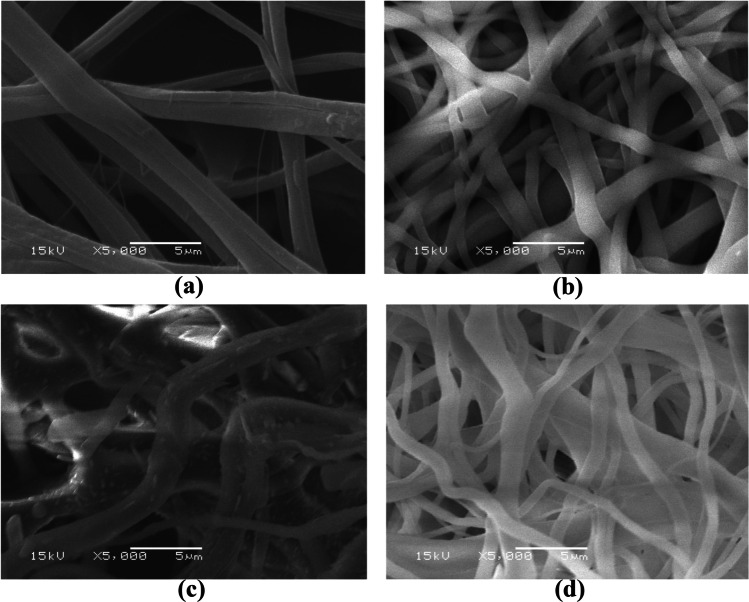
The SEM images were taken at 15 kV and magnification of 5000 × for **a** CA nanofiber mats, **b** CA/AC3.7, **c** CA/AC5.5, and **d** CA/AC6.7 composite nanofibers

Figure 1 (a, b, c, and d) show the SEM images of CA, CA/AC3.7, CA/AC5.5, and CA/AC6.7 composite mats prepared by electrospinning. The surface morphology of the prepared mats showed a randomly oriented bead-free matrix nanofiber network. The average fiber diameters of CA, CA/AC3.7, CA/AC5.5, and CA/AC6.7 composites were 495, 740, 745, and 759 nm, respectively, which were calculated as a mean value of 20 measurements using ImageJ software (Elmaghraby et al. 2022). It is clearly found that the average fiber diameter increases with increased AC content to CA nanofibers, as it enlarges the fiber diameter without destroying the fiber structures (Liu et al. 2020).

**Fig. 2:**
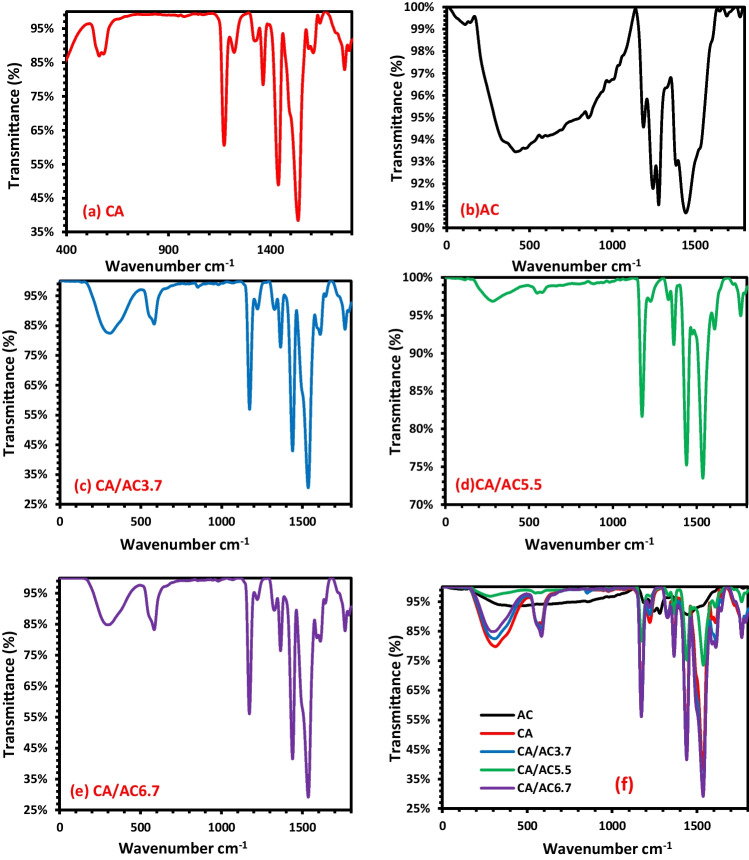
FT-IR analyses of **a** CA, **b** AC, **c** CA/AC3.7, **d** CA/AC5.5, and **e** CA/AC6.7 composite nanofibers; **f** represents image (a) to image (e) in one image for comparison

The FT-IR spectra of CA, AC, CA/AC3.7, CA/AC5.5, and CA/AC6.7 samples are given in Fig. 2. CA, CA/AC3.7, CA/AC5.5, and CA/AC6.7 samples exhibited same beaks at 3395 cm^−1^ due to the O–H, and beak at around 1737 cm^−1^ originates from C = O ester stretching vibration. The beaks at 1228 and 1368 cm^−1^ are due to the bending vibration of C–O and C–H groups, respectively. A strong beak at 1050 cm^−1^ was due to the stretching vibration of C–O–C in CA (Mohy Eldin et al. 2016). The FT-IR spectra of AC showed three peaks at 3477 cm^–1^ for O–H, 1741 cm^–1^ for C = O, and 1340 cm^–1^ for C–C vibrations. Clearly, by adding activated carbon to CA nanofiber mats, no change in the beaks was observed even if increasing activated carbon content.

**Fig. 3:**
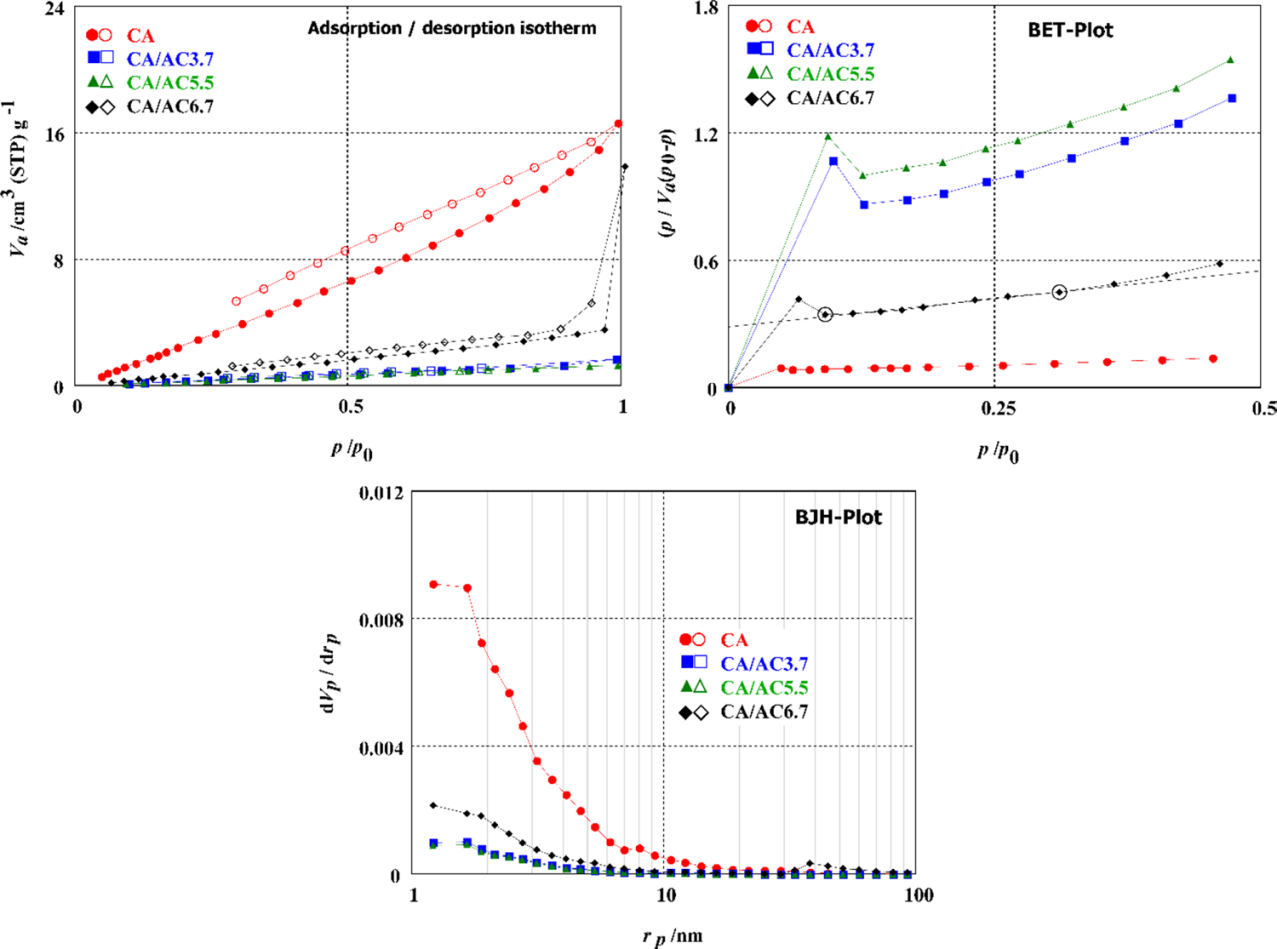
**a** Adsorption/desorption of N_2_ gas isotherms, **b** BET analysis, and **c** BJH analysis of CA, CA/AC3.7, CA/AC5.5, and CA/AC6.7 composite nanofiber.

According to the IUPAC classification, the adsorption–desorption curve of CA, AC, CA/AC3.7, CA/AC5.5, and CA/AC6.7 belonged to type V isotherm, as shown in Fig. 3 (Gregg and Sing 1982; Sing et al. 1985). The adsorption–desorption curve type V isotherm indicates that the adsorbent-adsorbate interactions are weak with mesoporous or microporous adsorbents. Because the isothermal loop deceleration does not stabilize at relative pressures that are close to the saturation vapor pressure, it is possible to deduce that mats have pores that are similar to slits. This can be inferred from the fact that the saturation vapor pressure is close to the relative pressure. The BET measurements and surface parameters of CA and CA/AC composite are detailed in the report that can be found in Table 1. These parameters include the surface area, average pore diameter, and pore volume. The surface area of the CA was found to be somewhat decreased with increased AC content in nanofibers and then increased with 6.7% AC load to CA, which may be related to the surface imperfections of the CA/AC composite. This was observed both before and after the addition of 6.7% AC load to CA. The largest surface area was 7.6913 m^2^/g for CA/AC6.7 composite, which was closed to the surface area of cellulose acetate nanofiber, which was 7.6522 m^2^/g. The surface area of CA/AC3.7, CA/AC5.5, and CA/AC6.7 composites were 6.744, 2.1374, and 7.6522 m^2^/g, respectively. The mean pore diameters (MPD) of CA/AC3.7, CA/AC5.5, and CA/AC6.7 composite mats were 1.5113, 5.9447, and 7.1716 nm, respectively.

**Table 1:** The CA/AC composite nanofiber was analyzed for its total surface area (TSA), total pore volume (TPV), and MPD

Sample	TSA (m^2^/g)	TPV (cm^3^/g)	MPD (nm)
CA	7.6522	0.0235	10.6380
CA/AC3.7	6.7442	0.0026	1.5113
CA/AC5.5	2.1374	0.0032	5.9447
CA/AC6.7	7.6913	0.0138	7.1716

**Fig. 4:**
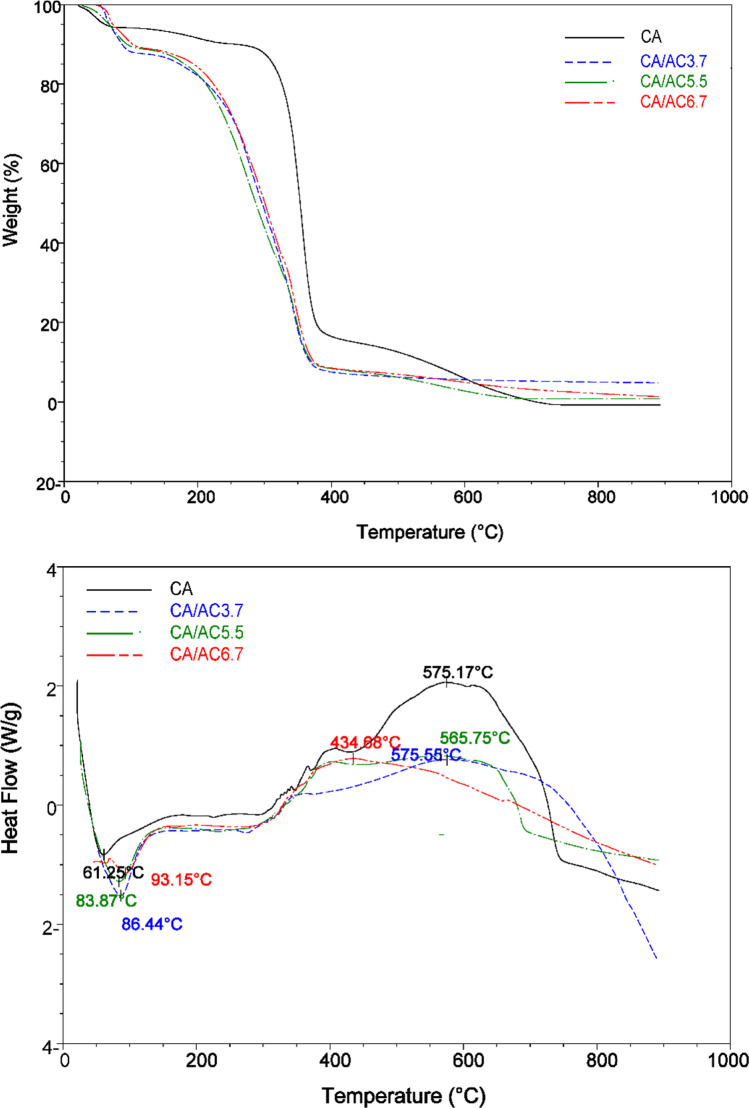
**a** TGA curves, **b** DSC curves of the CA, CA/AC3.7, CA/AC5.5, and CA/AC6.7 underflow of N_2_ (100 mL/min) using heating temperature from 50 to 900 °C.

The TGA curves of CA, CA/AC3.7, CA/AC5.5, and CA/AC6.7 composites are shown in Fig. 4 a. The fabricated composite nanofiber mat displayed two steps of thermal transition, the first at a low temperature between 50 and 150 °C with weight loss 10.32, 12.42, 10.68, and 10.71% for CA, CA/AC3.7, CA/AC5.5, and CA/AC6.7 composites, respectively. These were equivalent to moisture loss, whereas the second was at a higher temperature between 350 and 400 °C with weight loss of 73.01, 79.57, 80.47, and 80.13% for CA, CA/AC3.7, CA/AC5.5, and CA/AC6.7 composites, respectively, which represents the primary thermal degradation of cellulose acetate chains. In addition, the residues of CA/AC3.7, CA/AC5.5, and CA/AC6.7 composites after thermal decomposition at 900 °C were 16.67, 8.01, 8.85, and 9.16 wt%, respectively. The differential scanning calorimetry (DSC) technique is used for determining thermal transitions without weight change (El Nemr et al. 2021). It provides insight into a material’s phase shifts (melting point, boiling point, and fusion), heat capacity (*C*_*p*_), and glass transition temperatures by monitoring heat flow over temperature (Tg). DSC analysis of CA, CA/AC3.7, CA/AC5.5, and CA/AC6.7 composite nanofibers is shown in Fig. 4 b. CA, CA/AC3.7, CA/AC5.5, and CA/AC6.7 composite nanofibers displayed two peaks in DSC plot. All composite samples fabricated were exhibited a crystallization endo peak at a crystallization temperature of 61.07, 86.44, 83.87, and 93.15 °C for CA, CA/AC3.7, CA/AC5.5, and CA/AC6.7 composite nanofibers, respectively, and are due to sample water content loss. The exo melting peak represented a degradation temperature of CA, CA/AC3.7, CA/AC5.5, and CA/AC6.7 composite nanofibers were 575.17, 575.55, 565.75, and 434.68 °C, respectively that was attributed to the degradation of cellulose acetate. Figure 4 b shows clearly that by adding AC to hybrid mats, the observed maximum degradation temperatures of CA increased to 575.55 for CA/AC hybrid mats, respectively, as AC supports CA fiber. As revealed in SEM pictures, there is a point bonded structure in the hybrid mat due to the homogeneous mixing of CA and AC. For CA/AC composite nanofibers, a higher concentration of AC in the CA nanofibers resulted in a lower decomposition onset temperature.

### Water uptake

**Fig. 5:**
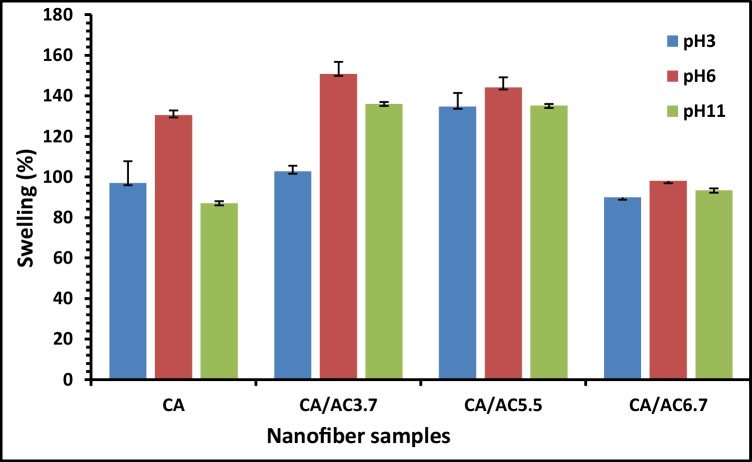
Results of the CA, CA/AC3.7, CA/AC5.5, and CA/AC6.7 composites’ equilibrium swelling at pH media (3, 6, and 11)

The swelling behavior of a CA/AC nanofiber composite is vital in the sorption application, as it allows the absorption of body fluid within the nanofiber giving a proof for good sorption properties (Tan et al 2020). The swelling performance of CA/AC nanofiber composites was studied at different pH media (3, 6, and 11 following reported data by Shi et al. (2014) and Bhandari et al. (2017)), as shown in Fig. 5. The swelling test was done for 24 h, and the maximum swelling ratio of CA membrane reached 130% at pH 6, and for the CA/AC composite nanofiber mats displayed higher values of swelling at the same pH (150.8, 144.11, and 97.93) for CA/AC3.7, CA/AC5.5, and CA/AC6.7, respectively. Subsequently, the highest swelling ratio was obtained at neutral and alkaline medium, whereas at pH 3 the swelling ratio was significantly low. Similar to what has been reported (Shi et al. 2014; Bhandari et al. 2017), the alkaline condition increased swelling, while the acidic condition decreased it. The swelling ratio of the pure CA nanofibers increases with adding AC content up to 5.5% AC which may be due to activated carbon allowing penetration of water molecules into the nanofiber network, which leads to increase the interspace between the polymer molecules, so the swelling ratio increases (Serag et al. 2018). However, by increasing AC content to 6.7%, the swelling ratio decreased, that is attributed to AC clogging the pores in fiber network and causing aggregations which hinders the water uptake process.

### ORC and sorption OSC

The oil retention capacity and adsorption of the CA, CA/AC3.7, CA/AC5.5, and CA/AC6.7 nanofiber mats are reported in Table 2. The maximum oil sorption capacity (OSC) for CA/AC nanofiber composite reached 9.06 g/g for heavy machine oil with CA/AC 3.7, which means that it can absorb more than 9 times of oil over its own weight. The best light machine oil sorption capacity was 5.8 g/g obtained by CA/AC5.5. CA/AC nanofiber composite showed a high oil retention capacity that reached 91.3% for heavy oil and 93% for light oil after 24-h dripping. It is clearly found that adding AC to CA mats increased the OSC and ORC. It was shown from the result, the sorption of HMO is slightly more than LMO, which is attributed to HMO being denser than LMO within the same volume unit. Generally, viscosity plays an essential role in enhancing the oil sorption process by boosting the adhered oil molecules on the adsorbent surface (Mohy Eldin et al. 2017). In contrast, LMO was more easily dropped out from steel mesh than HMO (Abdullah et al. 2010).

**Table 2:** ORC and OSC of the fabricated fiber samples CA, CA/AC3.7, CA/AC5.5, and CA/AC6.7 composites

Sample code	HMO		LMO	
	*Q*_*osc*_	*Q*_*orc*_	*Q*_*osc*_	*Q*_*orc*_
CA	8.23	91.3	4.45	80
CA/AC 3.7	9.06	91.3	5.59	92.2
CA/AC5.5	8.94	90.4	5.82	93.7
CA/AC6.7	8.23	91.3	5.34	92.8

### Batch adsorption experiments

CA/AC composite nanofibers were examined for their ability to absorb HMO and LMO from water. Different sorption parameters were studied, including sorbent dose, contact time, and oil thickness, to determine the high sorption capacity.

#### Effect of time

**Fig. 6:**
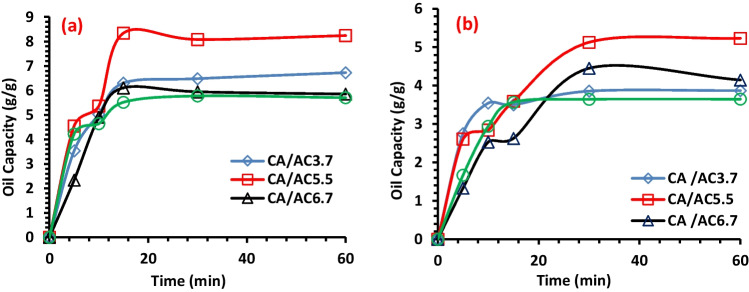
Impact of contact time on the OSC of **a** HMO and **b** LMO onto CA, CA/AC3.7, CA/AC5.5, and CA/AC6.7 composites

About 0.4 g of each fabricated composite fiber was tested for heavy and light machine oil sorption with 5-mm-oil thickness of HMO and 7-mm-oil thickness of LMO at different sorption times of (5, 10, 15, 30, and 60) min. Figure 6 displays the effect of sorption time on the OSC of HMO and LMO by CA/AC3.7, CA/AC5.5, and CA/AC6.7 composite mats. It is obviously shown from the results that the CA/AC nanofiber composite have a fast sorption rate during the first 5 min for HMO. The maximum sorption capacity was obtained within 15 min for HMO, and 30 min for LMO reached 8.3 and 5.5 g/, respectively, for CA/AC5.5 composite. In addition, the OSC somewhat increases with the contact time during the first 15 min; beyond that, the process for all of the adsorbents is quite slow. This can be explained by the fact that the oil on the exterior surface of the fibers can gently work its way into the hollow lumen. The findings of Thompson et al. (2010) and Wang et al. (2013) are in agreement with this observation.

#### Effect of sorbent dosage

**Fig. 7:**
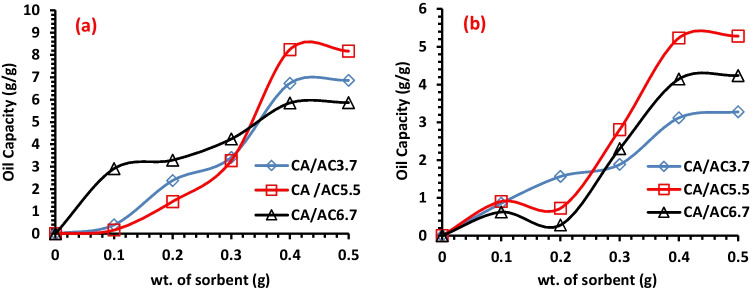
Impact of composite weight on the sorption capacity of **a** HMO and **b** LMO onto CA/AC3.7, CA/AC5.5, and CA/AC6.7 composite nanofibers

The quantity or mass used from CA/AC3.7, CA/AC5.5, and CA/AC6.7 composites were studied for the removal of HMO and LMO, as it considered an important parameter in industrial applications. Different sorbent doses of (0.1, 0.2, 0.3, 0.4, and 0.5 g) of each fabricated fiber were examined for oil sorption with 5-mm-oil thickness at 15 min of HMO and 7-mm-oil thickness at 30 min of LMO. Figure 7 investigates the HMO and LMO sorption capacity increase by increasing the sorbent dose from 0.1 to 0.5 g. The maximum sorption capacity for HMO reached 8.2 g/g by CA/AC5.5, while it reached 5.2 g/g by CA/AC5.5 for LMO. Increases in the number of exposed sorption sites that are available for the sorption process per unit gram of CA and CA/AC composites may be responsible for the outcomes that were observed (Liu et al. 2014). Also, it is essential to note that the highest values of the sorption capacity were recorded using which confirmed static oil sorption. Furthermore, the highest sorption capacity values, which were given by CA/AC5.5, indicate that adding activated carbon to CA mats increased the oil sorption capacity, but further increase of activated carbon cause aggregations which hinders the water uptake process.

#### Impact of oil spill thickness

**Fig. 8:**
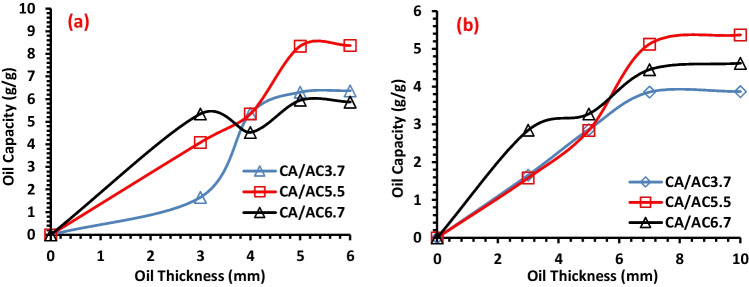
Impact of oil thickness layer on the sorption capacity of **a** HMO and **b** LMO onto CA/AC3.7, CA/AC5.5, and CA/AC6.7 composite nanofibers

The different oil layer thickness was studied on the sorption capacity of CA/AC3.7, CA/AC5.5, and CA/AC6.7 composite nanofibers. The sorption experiments were done as 0.4 g of the fabricated fiber was added to the oil/seawater system with different thicknesses of HMO (3, 4, 5, and 6) mm for 15 min and LMO (3, 5, 7, and 10) mm for 30 min. Figure 8 shows the effect of HMO and LMO thicknesses on the sorption capacity, as there is an increase in the sorption capacity with increasing the thickness of HMO and LMO. Then, further increases of oil amount up to 5 mm of HMO thickness layer and 7 mm of LMO thickness layer; the sorption capacity tends to be constant at constant seawater (50 mL); this may be due to the sorbent sites being saturated with oil spills. The maximum sorption capacity for HMO reached 8.35 g/g, while for LMO reached 5.13 g/g by CA/5.5AC composite nanofibers. It accomplishes this by increasing the oil thickness, which supplies a key driving force to overcome all resistances of the oil between the aqueous and solid phases, ultimately resulting in an increase in oil uptake. This is done by means of the oil’s interaction with the aqueous phase. In addition, there is a correlation between a rise in the initial oil thickness and an increase in the number of collisions between the oil and the adsorbent, which in turn leads to an increase in the OSC (Omer et al.^2019^).

### Reusability

**Fig. 9:**
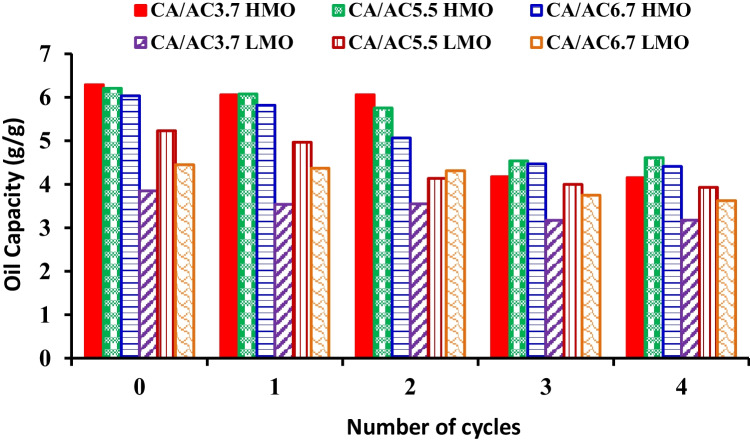
The reusability of CA/AC3.7, CA/AC5.5, and CA/AC6.7 for HMO/water and LMO/water systems (4 cycles)

The reusability is an essential factor for the selection of sorbent materials. The oil-saturated samples were recycled by using the centrifuge method and *n*-hexane extraction for further use in several sorption/desorption cycles. The OSC after regeneration of CA/AC3.7, CA/AC5.5, and CA/AC6.7 composite nanofibers for oil/seawater system are shown in Fig. 9, while Table 3 shows the reusability of CA/AC composites in static oil solution. The regeneration method showed easy reuse of sorbent materials where the fibers show higher oil uptake in the first cycle. Then the absorption capacity decreased, which can be attributed to the oil remaining in the fiber structure or due to the washing process causing deterioration of fiber structure, and the sorption capacity at the last two reusability cycles remained constant. Furthermore, the highest sorption capacity of oil/water solution for CA/AC composite was 8.3 and 5.2 g/g, which decreased to about 4.6 and 3.9 g/g for HMO and LMO, respectively. Thus, CA/AC composites are considered highly potential and promising oil-absorbing materials for oil spill cleanup (Teli and Valia 2013).

**Table 3:** The reusability of CA/AC3.7, CA/AC5.5, and CA/AC6.7 for static oil (4 cycles) for HMO and LMO

No. of cycles		0	1	2	3	4	0	1	2	3	4
Type of oil		HMO					LMO				
CA/AC3.7	OSC (g/g)	8.23	6.07	6.07	4.17	4.80	5.59	4.67	4.37	4.34	4.22
	St. dv	0. 081	0.062	0.071	0.043	0.052	0.093	0.085	0.063	0.069	0.078
	PL (%)	-	26.24	26.24	49.33	41.67	-	16.45	21.82	22.36	24.50
CA/AC5.5	OSC (g/g)	9.06	6.00	6.00	4.54	4.49	5.82	4.40	4.44	3.62	3.54
	St. dv	0. 088	0.052	0.065	0.067	0.059	0.082	0.074	0.069	0.053	0.063
	PL (%)	-	33.77	33.77	49.88	50.44	-	24.39	23.71	37.8	39.18
CA/AC6.7	OSC (g/g)	8.94	5.57	5.57	4.47	3.27	5.34	4.49	3.25	3.03	3.06
	St. dv	0.67	0.056	0.070	0.072	0.068	0.079	0.063	0.054	0.053	0.060
	PL (%)	-	37.69	37.69	50	63.42	-	15.91	39.13	43.25	42.69

*St. dv*. standard deviation, *PL (%)* percentage loss adsorption capacity.

### Adsorption isotherm

**Fig. 10:**
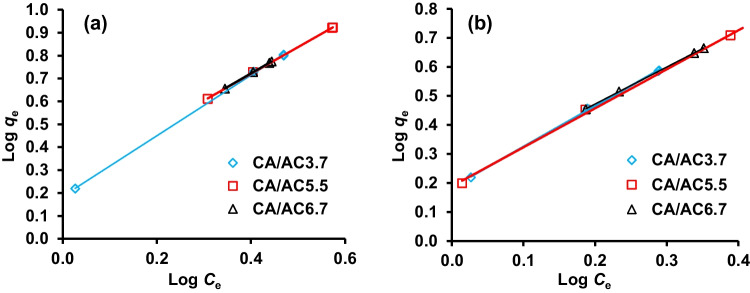
Freundlich model for adsorption of **a** HMO and **b** LMO over CA/AC3.7, CA/AC5.5, and CA/AC6.7 composite nanofibers

The adsorption isotherm behaviors are investigated for the connection that exists between the equilibrium concentrations of CA and CA/AC composites in the bulk and the oil amount at the solid surface. Langmuir, Freundlich, and Temkin isotherm equations were tested to investigate the correlation between the oil amount adsorbed and the oil concentration, which are the most widely accepted surface sorption models. The correlation coefficient (*R*^2^) was evaluated to determine the applicability of the isotherm equations. The result in Table 4 shows that the adsorption data of oil fitted with the Freundlich model better than another isotherm model, as represented by higher (*R*^2^) = 1 (Fig. 10). The Freundlich isotherm model studies the adsorption on heterogeneous surfaces and also suggests that the energy of sorption decreases exponentially when the sorption centers of an adsorbent are completed (El Nemr et al. 2010). The amount of oil adsorbed onto adsorbent in unit-balance-concern is represented by *K*_*F*_, and the deviation from the linearity of adsorption is represented by the heterogeneity factor (1/*n*). The value of *n* indicates the non-linearity degree between the concentration of solution and adsorption: where *n* = 1 indicates that the adsorption is linear; *n* < 1 represents that the adsorption process is chemical; *n* > 1 shows that the adsorption is a physical process (Kargi and Cikla 2006). The data in Table 4 describe that the adsorption of oil by CA/AC3.6, CA/AC5.5, and CA/AC6.7 was on heterogeneous surfaces with the interaction between adsorbed molecules and from the value of *n* > 1 means that the adsorption process is a favorable physical process.

**Table 4:** The isotherm model parameters for HMO and LMO adsorption by CA/AC3.7, CA/AC5.5, and CA/AC6.7 composites

Isotherm model	Isotherm parameters	HMO			LMO		
		CA/AC3.7	CA/AC5.5	CA/AC6.7	CA/AC3.7	CA/AC5.5	CA/AC6.7
Langmuir	*Q*_*m*_	10.30	36.36	26.88	6.56	9.19	13.46
	*K*_*a*_ × 10^3^	131.07	49.98	65.25	191.79	146.57	113.89
	*R*^2^	0.98	0.97	1.00	0.96	0.91	0.99
Freundlich	1/*n*	1.32	1.17	1.19	1.40	1.34	1.27
	*K*_*F*_	1.53	1.79	1.76	1.53	1.54	1.65
	*R*^2^	1.00	1.00	1.00	1.00	1.00	1.00
Tempkin	*A*	0.26	79.76	1.51	0.25	2.72	2.19
	*B*	0.22	0.14	0.16	0.27	0.40	0.21
	*R*^2^	1.00	1.00	1.00	0.99	1.00	1.00

#### Error function studies for best-fit IM

Error function equations were investigated to study the best-fit IM. Table 5 summarizes the data obtained from different error functions, as the lowest accuracy isotherm model is the Langmuir isotherm. The most fitted isotherm models are Freundlich and Tempkin isotherm models. Nevertheless, the error functions that were investigated produced diverse outcomes for each isotherm model. Therefore, the comparison of the isotherm models ought to be centered on each error function in its own right.

**Table 5:** Several alternative error functions are used to represent the isotherm model that best matches the experimental equilibrium data for CA/AC3.7, CA/AC5.5, and CA/AC6.7 composites

Type of machine oil	IM	APE%	*X*^2^	Hybrid	ERRSQ	MPSD	EABS	RMS
Heavy oil	Langmuir	2679.7	23,764	3002.3	97,302	8351.8	116.46	7624.1
	Freundlich	0.43	0.00	0.52	0.00	0.78	0.02	0.71
	Tempkin	96.18	5.10	1562	30.98	105.77	5.34	96.56
Light oil	Langmuir	1289.5	11,113	1378.5	97,555	3903.9	106.67	3563.8
	Freundlich	0.89	0.00	1.08	0.00	1.39	0.03	1.27
	Tempkin	97.17	8.98	409.60	96.85	106.55	9.19	97.27

### Adsorption kinetic studies

**Fig. 11:**
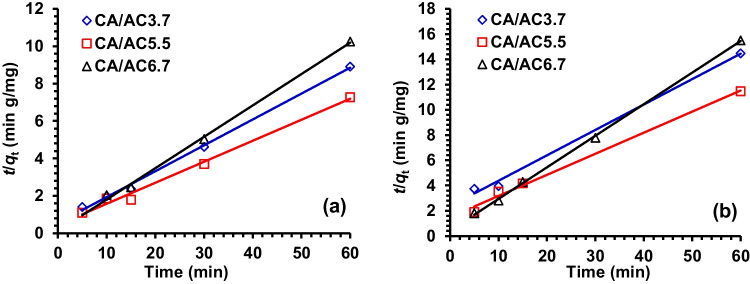
Plot *t*/*q*_t_ vs *t* of the PSO model for sorption of **a** HMO and **b** LMO by CA/AC3.7, CA/AC5.5, and CA/AC6.7 composites

The rate of adsorption was investigated using a number of different kinetic models. These models are essential for the design and modeling of adsorption processes because they control chemical reaction, the diffusion process, and mass transfer. PFO (Ho et al. 2000) and PSO (Zeldowitsch 1934) investigated the kinetics of the adsorption of oil onto CA and CA/AC composites, and the correlation coefficients (*R*^2^) expressed the conformity between experimental data and the model-predicted values. The PFO equation is used to represent the adsorption rate based on the adsorption capacity, which is often expressed by a plot of the values of log (*q*_*e*_ – *q*_*t*_) vs *t*, and the Lagergren parameters, *k*_1_ and *q*_e_, may be determined from the slope and intercept, respectively. According to the findings in Table 6, the experimental *q*_*e*_ acquired using the PFO model does not equal the estimated *q*_*e*_ derived from all of the data. Therefore, it is highly unlikely that the adsorption reaction will be a first-order reaction with a low correlation coefficient. While the PSO equation was used to describe the adsorption kinetic of CA/AC nanofiber composites, a plot of *t*/*q*_*t*_ vs *t* was used to illustrate the process. The slope and intercept of the line can be used to experimentally calculate the initial adsorption rate (*h*), the equilibrium adsorption capacity (*q*_*e*_), and the second-order constants (*k*_2_, g/mg min) (Fig. 11). According to the findings presented in Table 6, the computed correlations are less than 0.991 for the second-order kinetic model. As a result, the adsorption kinetics may very likely be more favorably represented by the PSO kinetic model for oil absorption. In addition, the values of *q*_*e*_ that were computed were, for the most part, equivalent to the *q*_*e*_ values that were measured experimentally, which demonstrated that the adsorption system conforms to the PSO kinetic model. The values of the initial adsorption rate (*h*), which represent the rate of initial adsorption, practically increased with the increase in initial oil concentrations. In contrast, the PSO rate constant (*k*_2_) decreased with an increase in oil amount (Table 6) (El Nemr et al. 2010).

**Table 6:** Data of PFO and PSO rate constants as well as the *q*_*e*_ values calculated and experimental for HMO and LMO adsorption onto CA/AC composites

Parameter	Type of machine oil	*q*_*e*_ (exp.)	PFO kinetic model			PSO kinetic model			
Samples			*k*_1_ × 10^3^	*q*_*e*_ (calc.)	*R*^2^	*k*_2_ × 10^3^	*q*_*e*_ (calc.)	*h*	*R*^2^
CA/AC3.7	Heavy oil	6.48	13.70	196.45	0.940	35.18	7.23	1.84	0.997
CA/AC5.5		8.08	0.72	40.53	0.111	27.67	8.90	2.19	0.991
CA/AC6.7		5.95	2.08	0.11	0.612	224.45	5.96	7.97	0.998
CA/AC5.5	Light oil	3.86	137.03	7.942	0.931	17.12	4.97	0.42	0.996
CA/AC3.7		5.13	115.15	1.549	0.662	18.38	5.99	0.66	0.994
CA/AC6.7		4.45	40.07	3.33	0.991	140.64	4.00	2.25	0.999

### Comparison study for removal of MO by CA/AC nanofiber composite with other adsorbents

The manufactured nanofiber composite had a maximum oil sorption capacity of 8.2 g/g. As shown in Table 7, the observed result was compared to earlier information reported in other literature. This comparison demonstrated that the manufactured CA/AC nanofiber composite demonstrates excellent efficiency for MO sorption, as it absorbs almost eight times as much oil as *Salvinia cucullata* Roxb and Slovenian woody material. Moreover, it absorbs forty times than rice husks. The manufactured CA/AC nanofiber composite might therefore be employed as an adsorbent to remove oil.

**Table 7:** Oil sorption capacities of various adsorbents given in the literature

Sorbent	Sorption capacity (g/g)	Reference
Carbonized cotton husks	5.1	Yang et al. (2020)
Slovenian woody material	1.3	Piperopoulos et al. (2020)
Rice husks after Pyrolysis (T: 480 °C, *t*: 3 h, under vacuum)	7.5	Angelova et al. (2011)
Sugarcane bagasse	3.2–5.3	Said et al. (2009)
Sugarcane bagasse esterification with stearic acid	1.3–3.2	Said et al. (2009)
Wool fibers	0.225, 5.56	Rajakovic et al. (2007)
Kapok fiber	0.827	Khan et al. (2004)
Rice husks	0.298	[Khan et al. (2004]
Coconut husk	0.058	Khan et al. (2004)
*Salvinia cucullata* Roxb	0.944	Khan et al. (2004)
Wood chips	0.343	Khan et al. (2004)
Reed canary grass (*Phalaris arundinacea*) screening with flax (*Linum usitatissimum* L.) and Hemp fiber (*Cannabis sativa* L.)	1.107	Khan et al. (2004)
Our study	8.2	-

## Conclusion

In conclusion, the CA/AC composites have a great potential to be used as efficient sorbent materials of HMO and LMO. Under optimum conditions, the CA/AC composite nanofiber mats absorbed more than their weight of HMO and LMO in 30 min. The maximum sorption capacity reached 8.3 and 5.5 g/g for HMO and LMO, respectively, obtained by CA/AC5.5 composite nanofibers. The CA/AC composite nanofibers showed higher oil uptake in the reusability test, and then its sorption capacity remained constant after the second cycle. Thus, CA/AC composite nanofibers are considered as a promising material for HMO and LMO adsorption from aquatic environment.

## Data Availability

The datasets used in this investigation are accessible for review upon request from the corresponding author of the paper.

## References

[CR1] Abdullah M, Rahmah AU, Man Z (2010). Physicochemical and sorption characteristics of Malaysian Ceibapentandra (L) Gaertn as a natural oil sorbent. J Hazard Mater.

[CR2] Aharoni C, Sparks DL (1991) Kinetics of soil chemical reactions – a theoretical treatment, in Sparks DL and Suarez DL (Eds). Rate of soil chemical processes. Soil Sci Soc Amr, Madison, WI. 1–18. 10.2136/sssaspecpub27.c1

[CR3] Aharoni C, Ungarish M (1977). Kinetics of activated chemisorption. Part 2. Theoretical models. J Chem Soc, Faraday Trans.

[CR4] Akkaya G, Ozer A (2005). Adsorption of acid red 274 (AR 274) on Dicranella varia: determination of equilibrium and kinetic model parameters. Process Biochem.

[CR5] Alaa El-Din G, Amer A, Malsh G, Hussein M (2017). Study on the use of banana peels for oil spill removal. Alexandria Eng J.

[CR6] Allen SJ, Gan Q, Matthews R, Johnson PA (2003). Comparison of optimized isotherm models for basic dye adsorption by kudzu. Bioresource Technol.

[CR7] Angel N, Guo L, Yan F, Wang H, Kong L (2020). Effect of processing parameters on the electrospinning of cellulose acetate studied by response surface methodology. J Agr Food Res.

[CR8] Angelova D, Uzunov I, Uzunova S, Gigova A, Minchev L (2011). Kinetics of oil and oil products adsorption by carbonized rice husks. Chem Eng J.

[CR9] Antunes dos Santos AE, Vieira dos Santos F, Freitas KM, Pimenta LPS, Andrade LO, Marinho TA, Fernandes de Avelar G, Bruna da Silva A, Ferreira RV (2021). Cellulose acetate nanofibers loaded with crude annatto extract: preparation, characterization, and in vivo evaluation for potential wound healing applications. Mater Sci Eng C.

[CR10] Ao C, Zhao J, Li Q, Zhang J, Huang B, Wang Q, Gai J, Chen Z, Zhang W, Lu C (2020). Biodegradable all-cellulose composite membranes for simultaneous oil/water separation and dye removal from water. Carbohyd Polym.

[CR11] Bhandari J, Mishra H, Mishra PK, Wimmer R, Ahmad FJ, Talegaonkar S (2017). Cellulose nanofiber aerogel as a promising biomaterial for customized oral drug delivery. Int J Nanomed.

[CR12] Choi HY, Bae JH, Hasegawa Y, An S, Kim IS, Lee H, Kim M (2020). Thiol-functionalized cellulose nanofiber membranes for the effective adsorption of heavy metal ions in water. Carbohydr Polym.

[CR13] Darmanin T, Guittard F (2015). Superhydrophobic and superoleophobic properties in nature. Mater Today.

[CR14] de Almeida DS, Duarte EH, Hashimoto EM, Turbiani FRB, Muniz EC, de Souza PRS, Gimenes ML, Martins LD (2020). Development and characterization of electrospun cellulose acetate nanofibers modified by cationic surfactant. Polym Testing.

[CR15] Dhaka A, Chattopadhyay P (2021). A review on physical remediation techniques for treatment of marine oil spills. J Environ Manage.

[CR16] Dong T, Xu G, Wang F (2015). Oil spill cleanup by structured natural sorbents made from cattail fibers. Ind Crops Prod.

[CR17] El Nemr A, Ragab S (2018). Acetylation of cotton-Giza 86 cellulose using MnCl_2_ as a new catalyst and its application to machine oil removal. Environ Process.

[CR18] El Nemr A, El-Sikaily A, Khaled A (2010). Modeling of adsorption isotherms of methylene blue onto rice husk activated carbon. Egypt J Aquat Res.

[CR19] El Nemr A, Moneer AA, Ragab S, El Sikaily A (2016). Distribution and sources of *n*-alkanes and polycyclic aromatic hydrocarbons in shellfish of the Egyptian Red Sea coast. Egypt J Aquat Res.

[CR20] El Nemr A, Eleryan A, Mashaly M, Khaled A (2021). Rapid synthesis of cellulose propionate and its conversion to cellulose nitrate propionate. Polym Bull.

[CR21] Elmaghraby NA, Omer AM, Kenawy ER, Gaber M (2022). El Nemr A (2022) Electrospun composites nanofibers from cellulose acetate/carbon black as efficient adsorbents for heavy and light machine oil from aquatic environment. J Iran Chem Soc.

[CR22] Freundlich HMF (1906). Über die adsorption in lösungen, Zeitschrift für PHysikalische. Chemie (Leipzig).

[CR23] Gregg SJ, Sing KSW (1982) Adsorption, surface area and porosity, 2nd ed. Academic Press, London. 10.1002/bbpc.19670710837

[CR24] Ho YS, Mckay G, Wase DAJ, Foster CF (2000). Study of the sorption of divalent metal ions on to peat. Ads Sci Technol.

[CR25] Ho YS, Chiu WT, Wang CC (2005) Regression analysis for the sorption isotherms of basic dyes on sugarcane dust. Bioresour Technol 96:1285–1291 10.1016/j.biortech.2004.10.021 15734316

[CR26] Jatoi AW, Kumar P, Kim IS, Ni QQ (2019). Sonication induced effective approach for coloration of compact polyacrylonitrile (PAN) nanofibers. Ultrason Sonochem.

[CR27] Jatoi AW, Ogasawara H, Kim IS, Ni Q-Q (2020). Cellulose acetate/multi-wall carbon nanotube/Ag nanofiber composite for antibacterial applications. Mater Sci Eng C.

[CR28] Kargi F, Cikla S (2006). Biosorption of zinc(II) ions onto powdered waste sludge (PWS): kinetics and isotherms. Enzyme Microb Technol.

[CR29] Khan E, Virojnagud W, Ratpukdi T (2004). Use of biomass sorbents for oil removal from gas station runoff. Chemosphere.

[CR30] Khan HW, Moniruzzaman M, Nasef MME, Khalil MAB (2020). Ionic liquid assisted cellulose aerogels for cleaning an oil spill. Matr Today: Proc.

[CR31] Lagergren S (1898). Zur theorie der sogenannten adsorption geloster stoffe. Kungliga Svenska Vetenskapsakademiens Handlingar.

[CR32] Langmuir I (1916). The constitution and fundamental properties of solids and liquids. J Amr Chem Soc.

[CR33] Liu F, Ma M, Zang D, Gao Z, Wang C (2014). Fabrication of superhydrophobic / superoleophilic cotton for application in the field of water / oil separation. Carbohydr Polym.

[CR34] Liu F, Li X, Wang L, Yan X, Ma D, Liu Z, Liu X (2020). Sesamol incorporated cellulose acetate-zein composite nanofiber membrane: an efficient strategy to accelerate diabetic wound healing. Inter J Biol Macromol.

[CR35] Long S, Feng Y, Liu Y, Zheng L, Gan L, Liu J, Zeng X, Long M (2021). Renewable and robust biomass carbon aerogel derived from deep eutectic solvents modified cellulose nanofiber under a low carbonization temperature for oil-water separation. Sep Pur Tech.

[CR36] Longhinotti E, Pozza F, Furlan L, Sanchez MDND, Klug M, Laranjeira MCM, Favere VT (1998). Adsorption of anionic dyes on the biopolymer chitin. J Brazil Chem Soc.

[CR37] Ma W, Zhang M, Liu Z, Kang M, Huang C, Fu G (2019). Fabrication of highly durable and robust superhydrophobic-superoleophilic nanofibrous membranes based on a fluorine-free system for efficient oil/water separation. J Membrane Sci.

[CR38] Martins LS, Zanini NC, Maia LS, de Souza AG, Barbosa RFS, Rosa DS, Mulinari DR (2021). Crude oil and S500 diesel removal from seawater by polyurethane composites reinforced with palm fiber residues. Chemosphere.

[CR39] MohyEldin MS, AbdElmageed MH, Omer AM, Tamer TM, Yossuf ME, Khalifa RE (2016). Development of novel phosphorylated cellulose acetate polyelectrolyte membranes for direct methanol fuel cell application. Int J Electrochem Sci.

[CR40] MohyEldin MS, Ammar YA, Tamer TM, Omer AM, Ali AA (2017). Development of low-cost chitosan derivatives based on marine waste sources as oil adsorptive materials: I. preparation and characterization. Desalin Water Treat.

[CR41] Nasir M, Subhan A, Prihandoko B, Lestariningsih T (2017). Nanostructure and property of electrospun SiO_2_-cellulose acetate nanofiber composite by electrospinning. Energy Procedia.

[CR42] Nasseh N, Barikbin B, Taghavi L, Nasseri MA (2019). Adsorption of metronidazole antibiotic using a new magnetic nanocomposite from simulated wastewater (isotherm, kinetic and thermodynamic studies). Composites Part b: Eng.

[CR43] El Nemr A (2005) Petroleum contamination in warm and cold marine environment. Nova Science Publishers, Inc. Hauppauge New York. [ISBN 1–59454–615–0] 150pp.

[CR44] Ng JCY, Cheung WH, McKay G (2002). Equilibrium studies of the sorption of Cu(II) ions onto chitosan. J Coll Interf Sci.

[CR45] Omer AM, Khalifa RE, Tamer TM, Elnouby M, Hamed AM, Ammar YA, Ali AA, Gouda M, MohyEldin MS (2019). Fabrication of a novel low-cost superoleophilic nonanyl chitosan-poly (butyl acrylate) grafted copolymer for the adsorptive removal of crude oil spills. Int J Biol Macromol.

[CR46] Pearce CI, Lioyd JR, Guthrie JT (2003). The removal of color from textile wastewater using whole bacterial cells: a review. Dyes Pigments.

[CR47] Piperopoulos E, Calabrese L, Mastronardo E, Proverbio E, Milone C, (2020) Sustainable reuse of char waste for oil spill recovery foams. Water Air Soil Pollut. 231. 10.1007/s11270-020-04671-2

[CR48] Porter JF, McKay G, Choy KH (1999). The prediction of sorption from a binary mixture of acidic des using single-and mixed-isotherm variants of the ideal adsorbed solute theory. Chem Eng Sci.

[CR49] Rajakovic V, Aleksic G, Radetic M, Rajakovic L (2007). Efficiency of oil removal from real wastewater with different sorbent materials. J Hazard Mater.

[CR50] Sabir A, Islam A, Shafiq M, Shafeeq A, Butt MTZ, Ahmad NM, Jamil T (2015). Novel polymer matrix composite membrane doped with fumed silica particles for reverse osmosis desalination. Desal.

[CR51] Sabir A, Shafiq M, Islam A, Jabeen F, Shafeeq A, Ahmad A, Jamil T (2016). Conjugation of silica nanoparticles with cellulose acetate/polyethylene glycol 300 membrane for reverse osmosis using MgSO_4_ solution. Carbohydr Polym.

[CR52] Said AEAA, Ludwick AG, Aglan HA (2009). Usefulness of raw bagasse for oil absorption: a comparison of raw and acylated bagasse and their components. Bioresour Technol.

[CR53] Salem DMSA, Morsy FA-EM, El Nemr A, El-Sikaily A, Khaled A (2014). The monitoring and risk assessment of aliphatic and aromatic hydrocarbons (PAHs) in sediments of the Red Sea. Egypt Egypt J Aquat Res.

[CR54] Salihu G, Goswami P, Russell S (2012). Hybrid electrospun nonwovens from chitosan/cellulose acetate. Cellulose.

[CR55] Satapathy M, Varshney P, Nanda D, Panda A, Mohapatra SS (2017). Fabrication of superhydrophobic and superoleophilic polymer composite coatings on cellulosic filter paper for oil-water separation. Cellulose.

[CR56] Serag E, El Nemr A, Fathy SA, Abdel Hamid FF, El-Maghraby A (2018). A novel three dimensional carbon nanotube-polyethylene glycol-polyvinyl alcohol nanocomposite for Cu(II) removal from water. Egypt J Aquat Biol Fish.

[CR57] Serag E, El-Maghraby A, El Nemr A (2022). Recent developments in the application of carbon based nanomaterials in implantable and wearable enzyme-biofuel cells. Carbon Lett.

[CR58] Shi X, Zheng Y, Wang G, Lin Q, Fan J (2014). pH and electroresponse characteristics of bacterial cellulose nanofiber/sodium alginate hybrid hydrogels for dual controlled drug delivery. RSC Adv.

[CR59] Sing KSW, Everett DH, Haul RAW, Moscou L, Pierotti RA, Rouquerol J, Siemieniewska T (1985). Reporting physisorption data for gas/solid interface with special reference to the determination of surface area and porosity. Pure Appl Chem.

[CR60] Tan H-L, Kai D, Pasbakhsh P, Teow S-Y, Lim Y-Y, Pushpamalar J (2020). Electrospun cellulose acetate butyrate/polyethylene glycol (CAB/PEG) composite nanofibers: a potential scaffold for tissue engineering. Colloids Surf b: Biointerfaces.

[CR61] Teli MD, Valia SP (2013). Acetylation of banana fibre to improve oil absorbency. Carbohydr Polym.

[CR62] Tempkin MJ, Pyzhev V (1940). Acta Physiochim. URSS.

[CR63] Thompson NE, Emmanue GC, Adagadzu KJ, Yusuf NB (2010) Sorption studies of crude oil on acetylated rice husks. Arch Appl Sci Res 2(5):142–151. http://scholarsresearchlibrary.com/archive.html

[CR64] Wang XS, Qin Y (2005). Equilibrium sorption isotherms for of Cu^2+^ on rice bran. Process Biochem.

[CR65] Wang J, Zheng Y, Kang Y, Wang A (2013). Investigation of oil sorption capability of PBMA/SiO_2_ coated kapok fiber. Chem Eng J.

[CR66] Wang W, Lin J, Cheng J, Cui Z, Si J, Wang Q, Peng X, Turng L-S (2020). Dual super-amphiphilic modified cellulose acetate nanofiber membranes with highly efficient oil/water separation and excellent antifouling properties. J Hazard Mater.

[CR67] Wang X, Nakane K (2021) Formation and morphological variation of electrospun cellulose acetate nano‐ fibers via dual-bath immersion electrospinning.Mater Lett 284(2):128968. 10.1016/j.matlet.2020.128968

[CR68] Wasim M, Sabir A, Shafiq M, Islam A, Jamil T (2017). Preparation and characterization of composite membrane via layer by layer assembly for desalination. Appl Surf Sci.

[CR69] Wasim M, Sabir A, Shafiq M, Khan RU (2019). Fractionation of direct dyes using modified vapor grown carbon nanofibers and zirconia in cellulose acetate blend membranes. Sci Total Environ.

[CR70] Yang M, Wang J, Chen Y, Gao J (2020). Biochar produced from cotton husks and its application for the adsorption of oil products. IOP Conf Ser Earth Environ Sci.

[CR71] Yue X, Zhang T, Yang D, Qiu F, Li Z, Zhu Y, Yu H (2018). Oil removal from oily water by a low-cost and durable flexible membrane made of layered double hydroxide nanosheet on cellulose support. J Cleaner Product.

[CR72] Zeldowitsch J (1934). Über den mechanismus der katalytischen oxidation von CO and MnO_2_. Acta Physicochim URSS.

